# Expression and Localization of Mitochondrial Ferritin mRNA in Alzheimer's Disease Cerebral Cortex

**DOI:** 10.1371/journal.pone.0022325

**Published:** 2011-07-20

**Authors:** Ligang Wang, Hongkuan Yang, Shiguang Zhao, Haruhisa Sato, Yoshihiro Konishi, Thomas G. Beach, Essam Mohamed Abdelalim, Naomi J. Bisem, Ikuo Tooyama

**Affiliations:** 1 Molecular Neuroscience Research Center, Shiga University of Medical Science, Otsu, Japan; 2 Department of Neurosurgery, 1st Affiliated Hospital, Harbin Medical University, Harbin, China; 3 Department of Pathology, Marutamachi Hospital, Kyoto, Japan; 4 Department of Clinical Research, Nishi-tottori National Hospital, Tottori, Japan; 5 Civin Laboratory for Neuropathology, Banner Sun City, Arizona, United States of America; Mental Health Research Institute of Victoria, Australia

## Abstract

Mitochondrial ferritin (MtF) has been identified as a novel ferritin encoded by an intron-lacking gene with specific mitochondrial localization located on chromosome 5q23.1. MtF has been associated with neurodegenerative disorders such as Friedreich ataxia and restless leg syndrome. However, little information is available about MtF in Alzheimer's disease (AD). In this study, therefore, we investigated the expression and localization of MtF messenger RNA (mRNA) in the cerebral cortex of AD and control cases using real-time polymerase chain reaction (PCR) as well as *in situ* hybridization histochemistry. We also examined protein expression using western-blot assay. In addition, we used *in vitro* methods to further explore the effect of oxidative stress and β-amyloid peptide (Aβ) on MtF expression. To do this we examined MtF mRNA and protein expression changes in the human neuroblastoma cell line, IMR-32, after treatment with Aβ, H2O2, or both. The neuroprotective effect of MtF on oxidative stress induced by H_2_O_2_ was measured by MTT assay. The *in situ* hybridization studies revealed that MtF mRNA was detected mainly in neurons to a lesser degree in glial cells in the cerebral cortex. The staining intensity and the number of positive cells were increased in the cerebral cortex of AD patients. Real-time PCR and western-blot confirmed that MtF expression levels in the cerebral cortex were significantly higher in AD cases than that in control cases at both the mRNA and the protein level. Cell culture experiments demonstrated that the expression of both MtF mRNA and protein were increased by treatment with H2O2 or a combination of Aβ and H2O2, but not with Aβ alone. Finally, MtF expression showed a significant neuroprotective effect against H2O2-induced oxidative stress (*p*<0.05). The present study suggests that MtF is involved in the pathology of AD and may play a neuroprotective role against oxidative stress.

## Introduction

We and others have previously shown that iron and iron regulatory proteins are involved in the pathology of Alzheimer's disease (AD). Postmortem examinations of human brain tissue have demonstrated that iron concentrations are increased in the brains of AD patients compared to controls [1], [2], [3]. Iron [1], [4], [5] and iron regulatory proteins such as ferritin [3], transferrin [3], [6], melanotransferrin [6] and lactoferrin [7], [8] have been detected in senile plaques and/or neurofibrillary tangles, reportedly major sites for catalytic redox activity [9]. Excessive iron deposition may generate some of the oxidative stress know to occur in AD [4], [9], [10], [11], [12].

Mitochondria have a key role in iron metabolism because they are involved in synthesizing heme and various iron-sulfur (Fe-S) cluster-containing proteins [13], [14]. The last step in heme biosynthesis, the insertion of Fe^2+^ into protoporphyrin IX by ferrochelatase, occurs in the mitochondrial matrix [13]. Mitochondrial iron levels must be well regulated because an inadequate supply of iron would impair the metabolic and respiratory activities of the organelle [14], [15], [16], while excess “free” iron in mitochondria would promote the generation of harmful reactive oxygen species which may cause diseases such as sideroblastic anemia [15].

Recently, mitochondrial ferritin (MtF) has been identified as a novel ferritin encoded by an intronless gene on chromosome 5q23.1. This gene product has been localized to the mitochondria in humans [17]. MtF is expressed as a 30-kDa precursor that is targeted to mitochondria by a positively charged leader sequence. Proteolysis of the N-terminal leader sequence within the mitochondria forms a 22-kDa MtF subunit, which has high-sequence homology to H-type ferritin, including conservation of the ferroxidase center and activity [18], [19]. MtF has been detected at very low levels in iron storage organs such as the liver and spleen, but levels are high in the testes in which mitochondria are abundant [19], [20]. The distribution pattern of MtF suggests that MtF may play an important role in mitochondrial function.

The function of MtF has not been elucidated. However, several studies have implicated MtF in a number of neurological disorders such as Friedreich ataxia [21] and restless legs syndrome [22]. Although abnormalities of mitochondrial function and iron metabolism have been reported in AD, little information is available about MtF in the brains of AD patients. In this study, therefore, we investigated the expression and localization of MtF messenger RNA (mRNA) in the cerebral cortex of AD and control cases using real-time polymerase chain reaction (PCR) and *in situ* hybridization histochemistry. We also examined MtF protein expression in AD patient brain tissues using a western-blot assay. In addition, we examined the effect of β-amyloid peptide (Aβ 1–42) and H2O2 treatments on the expression of MtF mRNA and protein in a human neuroblastoma cell line, IMR-32. The neuroprotective effect of MtFt against oxidative stress induced by H_2_O_2_ was also examined.

## Materials and Methods

### Ethics statements

Human brain tissues were obtained from the Brain Donation Program at the Banner Sun Health Research Institute [23]. Written informed consent was obtained from all donors or their legal representatives. The Brain Donation Program has been approved by the Institutional Review Board of the Banner Health Corporation. The study was also approved by the ethics committee at Shiga University of Medical Science.

### Brain samples

Total RNA and protein were purified from the temporal cortex and cerebellum of eight sporadic AD cases (mean age ± S.D., 82.3±9.2 years) and eight control cases without neurological disease (mean age ± S.D., 85.1±9.7 years). Brain tissue from another three AD cases (mean age ± S.D., 83.3±3.8 years) and three control cases (mean age ± S.D., 73.7±13.0 years) were used for *in situ* hybridization. For Western blots, brain tissue from three AD cases (mean age ± S.D., 80.7±9.0 years) and three control cases (mean age ± S.D., 79.0±7.1 years) was used. The average postmortem delay for the AD and control cases was 2.62 and 2.71 hours, respectively.

### *In situ* hybridization

Human MtF cDNA was obtained from the plasmid, pCMV6-XL5 (OriGene Technologies, Inc., Rockville, MD). The entire MtF coding region was inserted into the pGEM-T Easy vector (Promega, Madison, WI, USA) by PCR using the following primers: 5′-GAACAGGACGACTGGGAAAGCG-3′; and 5′- AGAGCGTGCAATTCCAGCAACG-3′. After linearization with *SacII* and *Sal*I, digoxigenin-UTP labeled sense and antisense riboprobes were transcribed using SP6 and T7 RNA polymerases according to the manufacturer's protocols. The riboprobes were subsequently purified by ethanol precipitation.

*In situ* hybridization was used to examine MtF mRNA expression in the temporal cortices from three AD cases and three control cases. The tissue was processed following previously described methods [24], [25], [26]. Sections were treated for 10 min at room temperature with 10 µg/ml proteinase K in 10 mM Tris-HCl buffer (pH 8.0) containing 150 mM NaCl at 37°C, and then post-fixed with 4% paraformaldehyde in 0.1 M PBS at room temperature for 10 min. Sections were pre-hybridized for 2 h at 37°C in hybridization buffer (50% formamide, 5 x Denhardt's solution, 3 x saline/sodium citrate (SSC; 1x: 150 mM NaCl and 15 mM sodium citrate), 0.5 mg/ml yeast tRNA (Gibco BRL), and 0.5 mg/ml heat-denatured salmon sperm DNA (Wako Pure Chemicals Co., Osaka, Japan). Probes were diluted in hybridization buffer to a final concentration of 2 µg/ml and hybridized for 16 h at 60°C. After hybridization, the sections were washed briefly in pre-warmed 3 x SSC at 60°C, then rinsed for 2 h in pre-warmed 0.2 x SSC buffer at 60°C. Sections were then rinsed for 5 min in NT buffer (0.1 M Tris HCl, pH 7.5 and 150 mM NaCl ) at room temperature. Sections were blocked in 1% skim milk in NT buffer for 60 min, and incubated overnight at 4°C with alkaline phosphatase-labeled anti-digoxigenin antibody (1:200; Roche Diagnostics, Basel, Switzerland). After washing with NT buffer, signal was detected using the substrates nitroblue tetrazolium chloride (NBT) and 5-bromo-4-chloro-3-indolylphosphate p-toluidine salt (BCIP).

### Preparation of Aβ oligomers

We used Aβ-derived diffuse ligands (ADDLs) as typical Aβ oligomers. ADDLs are a mixture of Aβ oligomers including SDS-resistant 3–24 mers of Aβ [27], [28], [29], [30], [31]. ADDLs are widely used for AD research as they display neurotoxicity and have been detected in the cerebrospinal fluid of Alzheimer's patients [32].

Aβ oligomers were prepared as described previously [33], [34]. Briefly, synthetic Aβ 1–42 peptide (Peptide Institute, Osaka, Japan) was dissolved in HFIP to 1 mM and incubated for 1 hour at 37°C. After incubating on ice for 15 min, the HFIP was then removed by evaporation and the resulting peptide was stored at −20°C. Before use, the peptide film was re-suspended to 5 mM in dimethyl sulfoxide (DMSO) [35] followed by sonication for 15 min. Aβ 1–42 oligomers were prepared by diluting the solution in DMEM/Phenol Red-free Ham's F-12 medium without glutamine to a final concentration of 100 µM and then incubating for 24 h at 4°C [36]. The preparation was centrifuged at 16,000×g for 10 min at 4°C to remove insoluble aggregates, and the supernatant containing soluble oligomers was transferred into clean tubes. These preparations were used directly for cell treatments. The concentration of Aβ oligomers was determined using a Bio-Rad protein dye assay reagent (Hercules, CA, USA). A final concentration of 15 µM was used for cell treatment. Formation of Aβ oligomers was determined by silver staining and examination by electron microscope.

### Silver staining of Aß oligomers

Samples were diluted at 1∶2 with Tricine sample buffer (Bio-rad Laboratories, Hercules, CA) and were not heated before loading. Five microliters of each sample was run on a 15–20% precast polyacrylamide Tris-Tricine gel (Supersep Tricine gel; Wako, Osaka, Japan). After electrophoresis, the gel was stained using a silver staining kit (Nacalai Inc., Osaka, Japan).

### Electron microscopy examination for Aß oligomer formation

Two microliters of each sample was adsorbed onto a carbon- and Formvar-coated copper grid (200-mesh) by floating on a drop of sample solution. After washing with distilled water, the sample on the grid was negatively stained with 2% aqueous uranyl acetate, and then dried. Samples were then observed under a transmission electron microscope (H-7600; Hitachi, Japan) at 80 kV.

### Cell culture and treatment with ADDL, H2O2 and peroxynitrite

A human neuroblastoma cell line (IMR-32, purchased from ATCC, CRL-2468, Manassas, VA, USA) was used in this study. Cells were prepared in modified Eagles medium (MEM) supplemented with 10% fetal bovine serum (FBS), 1% Sodium pyruvate (Sigma, St. Louis, MO, USA), 1% L-glutamine (Sigma, St. Louis, MO, USA) and 1% penicillin/streptomycin (Nacalai Inc., Osaka, Japan) at 37°C in a humidified atmosphere of 5% CO_2_ and 95% air.

Three different treatments were investigated using IMR-32 cells cultured in 12-well plates: 1) treatment with 15 µM Aβ oligomers for 24 h; 2) treatment with 300 µM hydrogen peroxide H2O2, diluted from 30% hydrogen peroxide solution (Santoku Chemical Industries Co., LTD, Japan), for 30 min; 3) treatment with both 15 µM Aβ and 300 µM H2O2, and, 4) treatment with 10 µM peroxynitrite (WAKENYAKU Co., LTD, Japan) for 24 hours as a second method of inducing oxidative stress [37]. Peroxynitrite concentration was determined spectrophotometrically at 302 nm in 0.1 M NaOH (å_302 nm_ = 1670/M per cm). Decomposed peroxynitrite was obtained by incubating peroxynitrite in complete medium for 30 min at room temperature. For a negative control, cells were treated with an equal amount of DMSO to that used during preparation of the Aβ oligomers. Cells were subsequently incubated for a further 18 h before being assessed for viability or other end point measures. All experiments were performed in triplicate.

### Real-time PCR for human AD brain and *in vitro* experiments

MtF mRNA was detected by real-time PCR using a Light Cycler system (Roche Diagnostics K.K., Tokyo, Japan). The sense primer was 5′- GCTCTATGCGTCCTACGTGTACTTGT-3′, and the antisense primer was 5′-TCCTGTTCCGGCTTCTTGAT-3′. Real-time PCR analysis for β-actin mRNA was also employed to assess the variability of mRNA content. The results were analyzed using the LightCycler Software second derivative maximum method (Roche Diagnostics). Relative quantification of mRNA was performed based on fluorescence measurements in comparison with a standard curve that was generated during the course of each PCR run: values were normalized to the β-actin mRNA levels in each sample. All experiments were performed independently at least three times. For the *in vitro* experiments, the detection of MtF mRNA expression by real-time PCR was the same as for the human AD brain real-time PCR assay as described above. Statistical significance was assessed using the one-way ANOVA test. Significance was set at *p*<0.05.

### Western-blot analysis of MtF

Human brain samples for western-blot were collected and treated as previously described [8]. Cells were lysed with RIPA buffer (50 mM Tris [tris (hydroxymethyl) aminomethane]-HCl, 150 mM NaCl, 1% Nonidet P-40, 0.5% sodium deoxycholate, and 0.1% sodium dodecyl sulfate [SDS]). Protein content was determined using the Bio-Rad protein dye assay reagent (Hercules, CA, USA). Lysates containing equal amounts of protein were heated for 3 min at 95°C in Laemmli loading buffer and resolved by SDS-polyacrylamide gel electrophoresis (SDS-PAGE). They were then transferred to a polyvinylidene difluoride membrane (Immobilon-P, Nippon Millipore Ltd., Tokyo, Japan). BioRad molecular weight markers (Precision Plus, Nippon BioRad Laboratories, Tokyo, Japan) were used. The blots were blocked by incubation for 1 hour with 5% nonfat milk in Tris-buffered saline (TBS) containing 0.1% Tween 20 (TBST) and were hybridized overnight at 4°C with anti-MtF rabbit polyclonal IgG 1∶1000 (A93251Hu, Uscn, Wuhan, China), or anti-actin mouse monoclonal IgG 1∶1000 (MAB1501, Chemicon International Inc., Billerica, MA, USA). After washing 3 times for 10 minutes each with TBST, the blots were incubated for 1 hour with horseradish peroxidase-linked anti-rabbit IgG 1∶10,000 (ImmunoPure, Pierce, Rockford, USA). After extensive washing with 25 mM TBST, labeling was visualized by chemiluminescence using ECL western blotting detection reagents (SuperSignal West Pico, Thermo Scientific, Rockford, IL).

### Transfecting the MtF-expressing plasmid into IMR-32 cells

The entire coding region of MtF was subcloned into the pGEM-T Easy vector as mentioned above. This produced a plasmid containing a 768-bp fragment which encompassed the entire human MtF sequence, and included a *Sal*I site at the 5′ end and a *SacII* site downstream of the stop codon at the 3′ end of the MtF gene. The 768-bp *Sal*I/*SacII* insert was excised and subcloned into the *Sal*I/*SacII* site of the pEGFP-N1 expression vector (CLONTECH Laboratories, Inc., Palo Alto, CA). Correct constructs were confirmed by sequencing.

IMR-32 cells were transiently transfected with the expression construct using the FuGENE HD transfection reagent (Roche Diagnostics, Mannheim, Germany). Briefly, 5×10^5^ or 1×10^4^ cells were plated on 60 mm dishes or on 96-well plates respectively and transfected according to the manufacturer's instructions. Cells were maintained in modified Eagles medium (MEM) supplemented with 10% fetal bovine serum (FBS), 1% Sodium pyruvate (Sigma, St. Louis, MO, USA), 1% L-glutamine (Sigma, St. Louis, MO, USA) in a humidified atmosphere (5% CO_2_, 95% air) at 37°C. Experiments were performed 24 h after transfection and protein expression after transfection was detected using western-blotting.

### Assessment of cell viability

Culture cells were transfected as described above. Twenty-four hours after transfection, the medium was replaced with fresh medium with or without 300 µM H_2_O_2_ and incubated for 30 min. Cells were subsequently incubated in fresh medium for a further 18 h before being assessed for viability. Cell viability was measured by MTT assay. Briefly, MTT (500 µg/ml, Bioassay Systems, Hayward, CA, USA) was added to each well and incubated for 4 h at 37°C. The medium was aspirated and cells were lysed with DMSO. The absorbance at 570 nm was measured with a TECAN infinite M200 microplate reader (TECAN, Austria). Each experiment was repeated three times. Statistical significance was assessed using the one-way ANOVA test. Significance was set at *p*<0.05.

## Results

### *In situ* hybridization histochemistry

**Figure 1:**
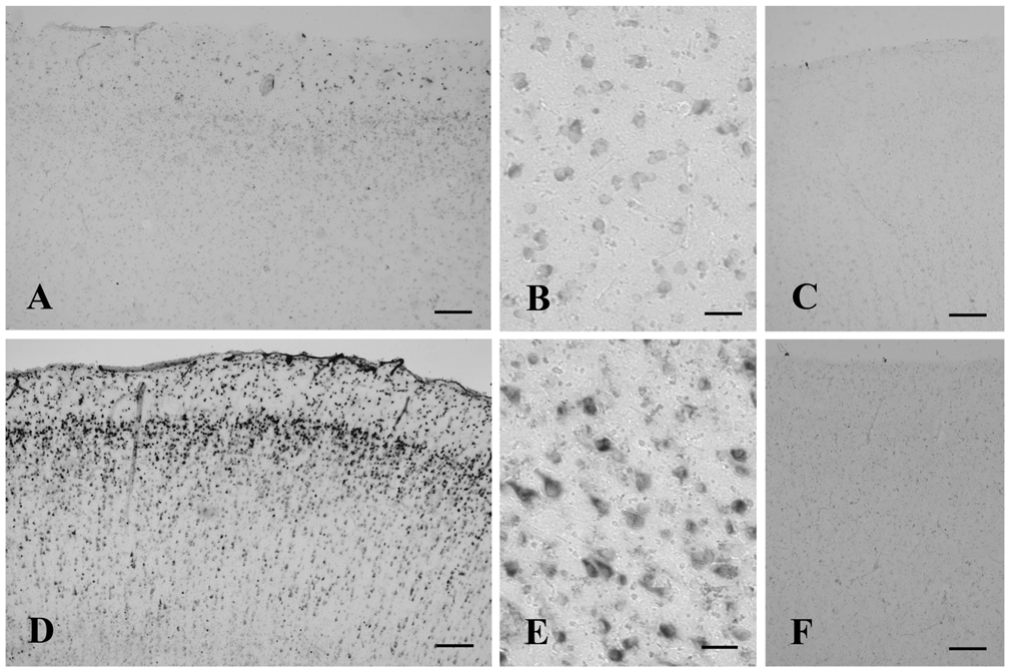
*In situ* hybridization histochemistry of the cerebral cortex of control (A–C) and Alzheimer's disease (AD) cases (D–F) using antisense (A, B, D, E) and sense (C, F) probes. Mitochondrial ferritin mRNA localizes mainly in neurons. Both the number and intensity of positive neurons increase in AD cases (D) compared to controls (A). Using sense probes (C and F), no signals are detected in the cortex. Bars  = 200 µm in A, C, D, F, and 50 µm in B, E.

Figure 1 shows the results of *in situ* hybridization histochemistry in the temporal cortex of a control (Fig. 1A, B, C) and an Alzheimer (Fig. 1D, E, F) case. Positive signals for MtF mRNA were detected in both control (Fig. 1 A) and AD (Fig. 1D) cases using the antisense probe. At a high magnification, positive signals were localized predominantly in neurons (Fig. 1E). The staining intensity in MtF-positive neurons was increased in AD cases (Fig. 1E) compared to controls (Fig. 1B). The sense probe did not generate any signal (Fig. 1C and F).

### Real-time PCR analysis using total RNA from human brain

**Figure 2:**
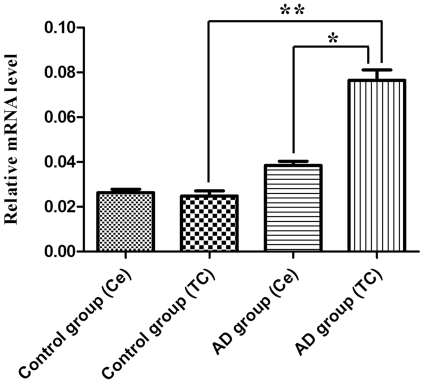
The expression levels of mitochondrial ferritin mRNA in the cerebral cortex of control and Alzheimer's disease (AD) patients and control cases. The expression in the temporal cortex (TC) of AD patients is significantly higher compared to that in control cases and cerebellum (Ce). Asterisk indicates significantly different to control (*p<0.05; **p<0.01).

Real-time PCR was used to compare the expression levels of MtF mRNA between AD and control cases (Fig. 2). When normalized to ß-actin, the mean expression levels of MtF mRNA in temporal cortex of control and AD cases were 0.023±0.003 (mean ± SEM, n = 8) and 0.075±0.009 (mean ± SEM, n = 8), respectively. The mean expression levels of MtF mRNA in the cerebellum of control and AD cases were 0.024±0.008 (mean ± SEM, n = 8) and 0.039±0.002 (mean ± SEM, n = 8), respectively. MtF mRNA levels in AD temporal cortices were significantly increased to 326% of control levels (*P<*0.01). There was no significant difference in MtF expression in the cerebellum between AD cases and controls.

### Determination of Aβ oligomer formation

**Figure 3:**
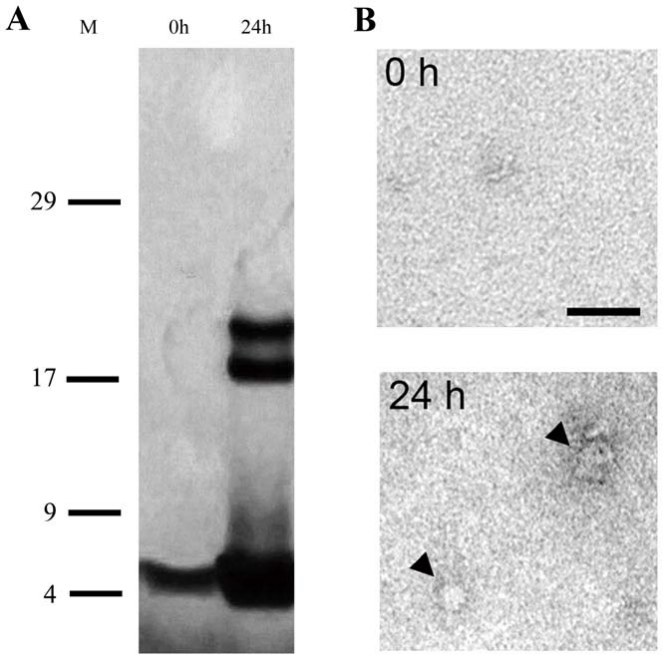
Formation of oligomeric Aβ1–42 assemblies. (A) Profiles of Aβ1–42 oligomers before (0 h) and after 24-h incubation (24 h) on SDS-PAGE followed by silver staining. M: Molecular weight markers. 0 h: Aβ1–42 preparation 0 h at 4°C. 24 h: Initial ADDL preparation 24 h later at 4°C. (B) Electrophoretic pattern of Aβ1–42 oligomeric preparations separated with centrifugal filters before electrophoresis. Representative photographs showing Aβ1–42 preparations before (0 h) and after incubation (24 h). Arrowheads in B indicate Aβ1–42 oligomers. Scale bar: 50 nm.

Supernatant proteins were separated on a Tris tricine gel using SDS-PAGE and visualized with a silver stain. SDS-PAGE analysis of oligomeric preparations before the 24-h incubation period showed no oligomeric species except for monomers (Fig. 3A; 0 h). After the 24-h incubation period, high molecular weight species between 17 and 25 kDa were apparent (Fig. 3A; 24 h). In these images, the uniform gray background is due to the silver staining process.

In addition, small and sparsely distributed spherical structures were detected by EM analysis after the 24-h incubation period (arrowheads in Fig. 3B).

### Real-time PCR analysis using total RNA from culture cells

**Figure 4:**
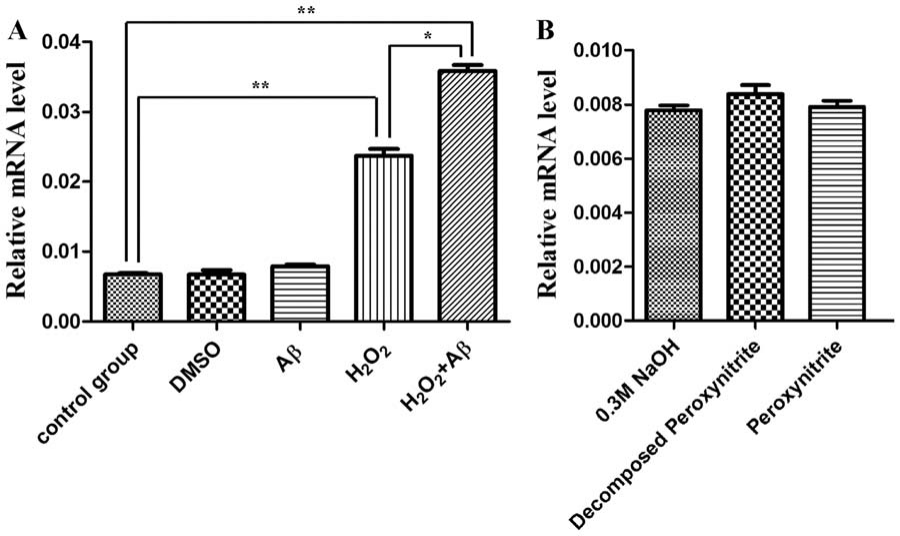
Mitochondrial ferritin mRNA expression levels in cultured cells. (A) Compared to the control, the MtF mRNA expression level is significantly high after H2O2 treatment but not after treatment with Aβ or DMSO. The group treated with both of H2O2 and Aβ displays the highest level of MtF mRNA expression among all groups. Asterisks and double asterisks indicate *p*<0.05 and *p*<0.01, respectively. (B) Results of real-time PCR analysis of MtF mRNA using total RNA from cultured cells in the peroxynitrite treatment group and the control groups. The MtF mRNA expression level was not changed significantly by peroxynitrite treatment.

Figure 4 A shows the results of real-time PCR analysis of MtF mRNA using total RNA from cultured cells in the H2O2 treatment group, the Aβ treatment group, the H2O2 plus Aβ treatment group, as well as the two control groups: DMSO treated and untreated cells. The MtF mRNA expression level was not changed by Aβ treatment but was significantly increased by H2O2 treatment (*P*<0.01). The results indicate that oxidative stress induces MtF expression in IMR32 cells. When Aβ neurotoxicity was added to oxidative stress, the expression of MtF mRNA was significantly accelerated. The MtF mRNA expression level in the combination group was the highest of all five groups (*p*<0.01 compared to control groups and *p*<0.05 compared to H2O2 treatment). DMSO treated groups showed no significant change in MtF expression.

Figure 4 B shows the results of real-time PCR analysis of MtF mRNA using total RNA from cultured cells in the peroxynitrite treatment group and the control groups. The MtF mRNA expression level was not changed significantly by peroxynitrite treatment. Neither the NaOH vehicle (0.3 M) nor the peroxynitrite decomposition products had a significant effect on MtF mRNA expression.

### Western blotting analysis of MtF

**Figure 5:**
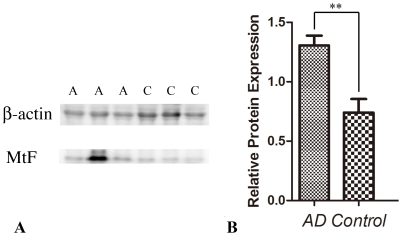
Western-blot analysis of MtF expression in human brain. (A) Three AD cases (lanes marked A) and three control cases (lanes marked C) were probed with anti β-actin antibody and anti MtF antibody. (B) When the expression level of MtF is normalized to β-actin, the expression level of MtF in the temporal cortex of AD patients is higher than in control cases (**p<0.01).

Figure 5 shows a western-blot analysis of MtF expression in human brain. The expression levels of MtF in the temporal cortex of AD patients (Fig.5 A, A) are higher than in control cases (*p<0.05*, Fig. 5 A, C).

**Figure 6:**
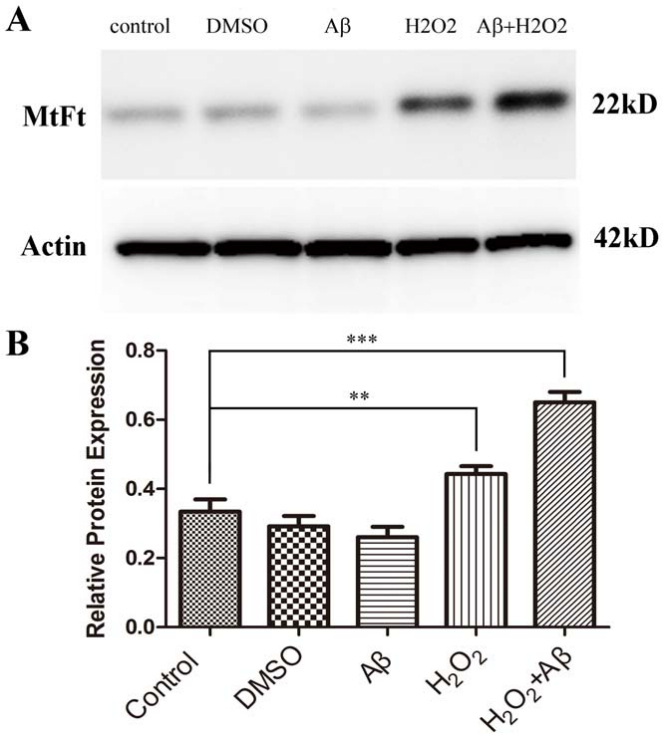
Western-blot analysis of MtF expression in each *in vitro* experiment group. (A) An immunoreactive band of approximately 22 kD probed with an anti-MtF antibody is present in all lanes. (B) When the expression level of MtF is normalized to β-actin, MtF expression after treatment with H_2_O_2_ or with a combination of Aβ and H_2_O_2_ was significantly greater than that in control cases (**p<0.01. *** p<0.001). However, treatment with Aβ or DMSO alone shows no significant effect on MtF expression.

Figure 6 shows the western-blot analysis of MtF protein expression in the H_2_O_2_ treatment group, the Aβ treatment group, the H_2_O_2_ plus Aβ treatment group, as well as the two control groups: DMSO treated and untreated cells. MtF protein expression after treatment with H_2_O_2_ or a combination of Aβ and H_2_O_2_ was significantly higher than in control groups. The combination group showed the highest MtF protein expression level of all groups. However, single treatment with Aβ or DMSO caused no significant effect on MtF protein expression. These results are in accordance with the real-time PCR results.

### MtF expression significantly rescues cell death after H_2_O_2_ treatment

**Figure 7:**
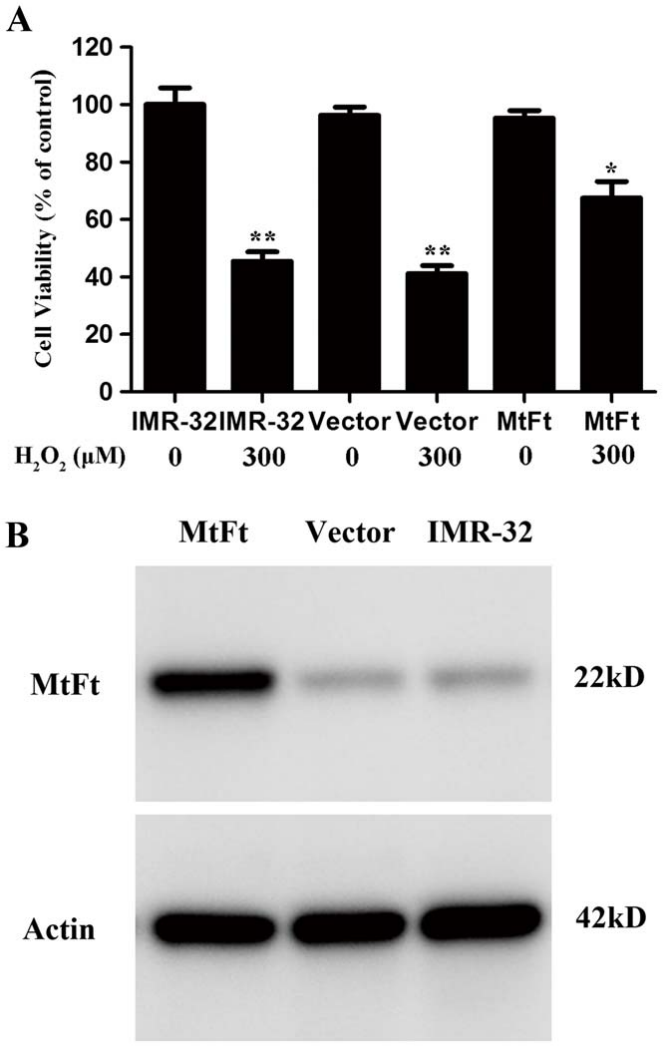
Effect of MtF expression on cell viability after treatment with H_2_O_2_. An MtF protein band exists with an apparent molecular mass of 22 kDa on SDS-PAGE; weak bands were detected in IMR-32 and vector-IMR-32 cells (B). Wild-type IMR-32 cells, empty vector transfectants (Vector-IMR-32), and MtF transfectants (MtF-IMR-32) were treated with 300 µM H_2_O_2_ for 30 min (A). Cell viability was measured by MTT assay. A remarkable decrease in the viability of IMR-32 and Vector- IMR-32 cells (about 50%; p<0.01, compared with control groups) was observed after treatment with 300 µM H2O_2_ for 30 min (A). The viability of MtF- IMR-32 cells under treatment decreased about 30%, however, the cell viability was much higher than in the control group (p<0.05). ** p<0.01 vs. non-treated cells; * p<0.05 vs. the H_2_O_2_-treated control cells.

In order to investigate the possible roles of MtF in neuroprotection, wild type IMR-32 cells (IMR-32), empty vector transfectants (vector-IMR-32) and MtF transfectants (MtF-IMR-32) were incubated with or without 300 µM H_2_O_2_ for 30 min. Figures 7A and B show the results of cell viability and western blots of MtF, respectively. Similar to previous reports [15], [17], [18], an MtF protein band with an apparent molecular mass of 22 kDa was detected by SDS-PAGE. Weak bands were detected in IMR-32 and vector-IMR-32 cells (Fig. 7B). A remarkable decrease in the viability of IMR-32 and Vector-IMR-32 cells (about 50%; *p<0.01*, compared with the control groups) was observed after treatment with 300 µM H_2_O_2_ for 30 min (Fig. 7A). The viability of MtF-IMR-32 cells decreased about 30% after H_2_O_2_ treatment, therefore cell viability was much higher than in control groups *(p<0.05*). These results show that overexpression of MtF reduces the rate of cell death after H_2_O_2_ treatment.

## Discussion

Using *in situ* hybridization histochemistry, we first demonstrated that MtF mRNA was localized predominantly in neurons in the temporal cortex of the human brain in both control and AD cases. Neuronal localization of MtF mRNA is in accordance with previous reports concerning localization of MtF protein in human substantia nigra [22] and mouse brain [20]. Interestingly, we demonstrated that the MtF mRNA signal intensity appeared to be increased in AD brains. In agreement with the *in situ* results, the quantitative PCR and western blot experiments showed that MtF mRNA and protein levels were increased in the temporal cortex of AD brains compared to controls. These results suggest that MtF may be involved in pathological processes in AD. More cases will be investigated in future studies to provide stronger evidence for a role of MtF in the progression of AD.

The cell culture experiments demonstrated that the expression of MtF was not altered by Aβ neurotoxicity or peroxynitrite treatment but was increased by H_2_O_2_ treatment. Peroxynitrite produces nitric oxide (NO) and can induce oxidative stress. However, treatment with peroxynitrite did not alter MtF expression in a similar way to H_2_O_2_. These results suggest that the increase in MtF expression may be specific to H_2_O_2_-induced oxidative stress. H_2_O_2_, a reactive oxygen species, is a by-product of normal cellular function and is produced by superoxide dismutase (SOD) and monoamine oxidase (MAO). Normally, H_2_O_2_ is broken down in brain by the actions of glutathione peroxidase and catalase; however, under certain pathophysiological conditions where excessive H_2_O_2_ is produced, cellular defenses can be overwhelmed and H_2_O_2_ may then provoke oxidative stress [38].

Interestingly, the present study demonstrated that MtF mRNA and protein expression were increased most by the combination of H2O2 and Aβ. However, there were no differences between Aβ treatment and the control group in terms of MtF mRNA and protein expression levels. Several previous studies have suggested a relationship between Aβ and oxidative stress induced by iron. It has been demonstrated that iron can facilitate the aggregation of Aβ and increase its toxicity [39], [40]. Iron has also been shown to induce the aggregation of hyperphosphorylated τ (tau), the major constituent of neurofibrillary tangles [5] and has been associated with senile plaques and neurofibrillary tangles in human AD brains [1], [4], [5]. Furthermore, it has been suggested that excessive iron can generate oxidative stress in AD [4], [9], [10], [11], [12].

The precise role of MtF in AD still remains unknown. However this and previous studies have raised several possibilities. Since Fe^2+^ displays a toxic effect on cells, the increased Fe^2+^ in AD brain tissues may be a cause of neuronal damage. In addition, Fe^2+^ can enhance the formation of Aβ oligomers and senile plaques [39], [40]. Because MtF has high-sequence homology to H-ferritin, including conservation of the ferroxidase center and activity [18], MtF is thought to oxidize the potentially toxic ferrous iron. In addition, MtF can reduce iron concentration in the cytoplasm. MtF has a positively charged leader sequence that provides it with a means to access the mitochondria [38]. Overexpression of MtF can draw iron from the cytosolic iron pool to the mitochondria and reduce cellular iron concentration [41], [42], [43]. Based on our *in vitro* results, it is likely that MtF may prevent cell injury and tissue damage, and therefore protect brain integrity, through antioxidant functions.

The neuroprotective effect of MtF is supported by the culture experiments showing that the overexpression of MtF decreased IMR-32cell death. However, even though there is an endogenous increase in MtFt upon incubation with H_2_O_2_, there is no rescue of cell viability in the same cells unless MtFt is expressed before H_2_O_2_ is added to the cells. This may be because H_2_O_2_-induced cell death occurs before MtFt can have its neuroprotective effect. Therefore, it appears that the MtF which is overexpressed before H_2_O_2_ addition has a greater protective effect than the endogenous MtF. Further study is needed to clarify this issue.

In summary, the present study has shown that MtF mRNA locates mainly in neurons and its expression is up-regulated in the cortex of AD patients. We have also demonstrated, through *in vitro* experiments, that this increase in MtF expression may be induced by H_2_O_2_-provoked oxidative stress rather than by Aβ. Moreover, MtF overexpression has a neuroprotective effect against oxidative stress induced by H_2_O_2_. These results indicate that MtF may be involved in the pathological process of AD.
